# Chondro-Protective Effects of Shikimic Acid on Osteoarthritis *via* Restoring Impaired Autophagy and Suppressing the MAPK/NF-κB Signaling Pathway

**DOI:** 10.3389/fphar.2021.634822

**Published:** 2021-07-27

**Authors:** Hongbo You, Rui Zhang, Lingying Wang, Qiyong Pan, Zekai Mao, Xiaojian Huang

**Affiliations:** ^1^ Department of Orthopedics, Tongji Hospital, Tongji Medical College, Huazhong University of Science and Technology, Wuhan, China; ^2^ Department of Radiation Oncology, Second Affiliated Hospital, School of Medicine, Zhejiang University, Hangzhou, China; ^3^ Department of Orthopaedics, First Affiliated Hospital of Zhengzhou University, Zhengzhou, China

**Keywords:** shikimic acid, osteoarthritis, autophagy, MMPs, MAPK, NF-κB

## Abstract

Osteoarthritis (OA) is a major cause of cartilage pain and limited mobility in middle-aged and elderly individuals. The degeneration of cartilage induced by inflammation and cartilage anabolic and catabolic disorder plays a key role in OA. Shikimic acid (SA), a natural ingredient extracted from *Illicium verum*, has been shown to exert notable anti-inflammatory effects in previous studies, suggesting its potential effects in the treatment of OA. In this study, we revealed that the pretreatment of SW1353 human chondrocytes with SA before interleukin 1β (IL-1β) stimulation effectively decreased the expression of inducible nitric oxide synthase (iNOS), cyclooxygenase (Cox)-2, matrix metalloproteinases (MMPs; MMP3 and MMP13), a disintegrin and metalloproteinase with thrombospondin motifs (ADAMTS)-5, type X collagen, and p62; increased the expression of type II collagen, ATG7, Beclin-1, and LC3; and increased the autophagic flux. Mechanistically, we found that SA suppressed the IL-1β-induced activation of the mitogen-activated protein kinase (MAPK) and nuclear factor-kappaB (NF-κB) pathways. Furthermore, the results of safranin O staining and toluidine blue staining of primary rat cartilage chondrocytes and a trauma-induced rat model of OA showed that SA alleviated progression of OA *in vivo*. Collectively, our research enhances understanding of the mechanism of protective effect of SA against the progression of OA, which involves amelioration of cartilage degeneration, thereby providing new evidence for the use of SA as a therapy to prevent the development of OA.

## Introduction

Osteoarthritis (OA) is a degenerative disease which is common in elderly individuals and is characterized by an inflammatory response and cartilage degeneration (Wieland et al., 2005; Martel-Pelletier et al., 2016). Although the number of people with OA has doubled in the past 60 years, the current treatment strategy for OA includes mainly nonpharmacological interventions with small to moderate therapeutic effects (McAlindon et al., 2014). There is no doubt that OA is becoming a burden for patients and healthcare systems. People with end-stage OA require joint arthroplasty, but statistics show that approximately 82% of total knee replacements (TKRs) last for only 25 years and require subsequent replacement revision, which is associated with additional risks (GBD 2016 DALYs and HALE Collaborators, 2017; Evans et al., 2019). Therefore, new strategies for OA treatment are needed. In recent years, studies have shown the potential effects of anti-inflammatory components derived from plants, such as artemisinin and salicin (Zhong et al., 2018; Gao and Zhang, 2019), in protecting against cartilage degeneration, suggesting that there are alternative strategies for OA treatment.

OA is a complex disease caused by multiple factors, but it is generally believed that inflammatory mediators, which can induce cartilage destruction by causing imbalance between anabolic and catabolic factors in chondrocytes, play vital roles in the development of OA. Among these inflammatory meditators, interleukin 1β (IL-1β) is regarded as a major participant (Goldring and Goldring, 2004). High levels of IL-1β are detected in the synovial fluid, subchondral bone, and cartilage of patients with OA (Smith et al., 1997). IL-1β suppresses local matrix synthesis and stimulates chondrocytes to release several proteolytic enzymes, such as matrix metalloproteinases (MMPs) and a disintegrin and metalloproteinase with thrombospondin motifs (ADAMTS)-5, which cleave matrix components (such as type II collagen, Col2) and lead to the degradation of the cartilage extracellular matrix (ECM) (Moos et al., 1999; Mengshol et al., 2000; Yang et al., 2017). The latest research shows that ablation of MMP13 with CRISPR/Cas9-based gene editing technology can alleviate OA (Zhao et al., 2019). Moreover, IL-1β stimulates the inflammatory response via overexpression of inducible nitric oxide synthase (iNOS) and cyclooxygenase (Cox)-2 (Gupta et al., 2008). The expression of type X collagen (Col 10) is one of the major characteristics of chondrocyte hypertrophy, which is followed by cartilage matrix degradation and vascular invasion (Moskowitz, 2000), and Col 10 might be a neoepitope biomarker of knee OA (He et al., 2019). Agents that antagonize IL-1β-associated OA-related factors might have a considerable efficacy for OA treatment.

Autophagy is a highly conserved catabolic process that maintains homeostasis of cellular energy regulation by mediating in the degradation of harmful or damaged materials. The formation of autophagosomes is an essential step in autophagy, and microtubule-associated protein light chain-3 (LC3) is a major constituent of autophagosomes, in which LC3I is converted to LC3II (Chen and Klionsky, 2011). Autophagosome formation involves a ubiquitin-like binding system in which ATG12 covalently binds to ATG5 and targets autophagic vesicles; this process can be mediated by ATG7 (Mizushima et al., 1998; Tanida et al., 1999). Beclin-1 is another key protein involved in this process. Previous studies have shown that Beclin-1 overexpression in cells stimulates autophagy (Tanida et al., 1999). Additionally, the protein p62/SQSTM1, one of the most well-characterized substrates of selective autophagy, directly interacts with LC3, and impairment of autophagy is accompanied by accumulation of p62 (Mizushima and Komatsu, 2011). Notably, via adenoviral monomeric RFP (mRFP)-GFP-LC3 transfection and confocal microscopy, we clearly observed autophagic flux (Zhou et al., 2018). An increasing number of studies have demonstrated that autophagy is associated with the pathogenesis of OA, and autophagy is considered a protective physiological process in normal cartilage (Caramés et al., 2010; Sasaki et al., 2012; Zhang et al., 2015). The level of autophagy is decreased after IL-1β-stimulation, and autophagy regulates changes in the expression of OA‐related biomarkers, including MMP13 (Xue et al., 2017; Zhang et al., 2018). Moreover, our previous studies have shown a relationship between cartilage chondrocyte catabolism and autophagy, with autophagy agonists alleviating and inhibitors intensifying the catabolism induced by IL-1β (Mao et al., 2018). Therefore, restoration of impaired autophagy may be a promising strategy for preventing chondrocyte degeneration in OA.

Shikimic acid (SA) is a compound found in many plants and is extracted from *Illicium verum* (Ghosh et al., 2012). SA has attracted attention due to its pharmacological safety and biological activity, and it has been widely used in the treatment of various diseases including gastric pain and skin inflammation (30). In RAW 264.7 cells, SA exerts anti-inflammatory effects by inhibiting lipopolysaccharide (LPS)-induced decrease in cell viability, nitrite accumulation, and proinflammatory cytokine production (Kolodziej et al., 2008). In addition, SA inhibits LPS-induced cellular proinflammatory cytokine production and attenuates mechanical hyperalgesia in mice (Estévez and Estévez, 2012; Rabelo et al., 2016). Furthermore, another investigation indicated that SA can inhibit NF-κB and MAPK pathways to inhibit osteoclastogenesis (Chen et al., 2018). However, the role of SA in cartilage degradation remains unclear. In this study, we evaluated whether SA exerts inhibitory effects on OA-related molecules in IL-1β-stimulated SW1353 human chondrocytes and rat cartilage.

## Materials and Methods

### Chemicals and Reagents

SA was purchased from Sigma-Aldrich (Merck KGaA; Darmstadt, Germany). Fetal bovine serum (FBS) was purchased from Glico-BRL (Gaithersburg, MD, United States). Recombinant human IL-1β, primary antibodies specific for iNOS, COX-2, MMP3, ATG7, Beclin-1, p62, LC3I/II, phosphorylated extracellular signal-regulated kinase (p-ERK), ERK, c-Jun N-terminal kinase (JNK), p-JNK, p38, p-p38, P65, and p-P65 were obtained from Cell Signaling Technology Inc. (Beverly, MA, United States) and used at a 1:1,000 dilution. Recombinant rat IL-1β was provided by R&D Systems (United States). Antibodies specific for MMP13 and type II collagen were purchased from Santa Cruz Biotechnology, Inc. (Santa Cruz, CA, United States) and used at a 1:500 dilution. Antibodies against glyceraldehyde-3-phosphate dehydrogenase (GAPDH), secondary antibodies, and a Cell Counting kit-8 (CCK-8) were obtained from Wuhan Boster Biological Technology Ltd. (Wuhan, China), and the antibodies were used at a 1:500 dilution.

### Cells Culture

All experiments involving animals were conducted according to the Guidelines of the Animal Care and Use Committee for Teaching and Research of Huazhong University of Science and Technology. Four-week-old Sprague Dawley rats were obtained from the Laboratory Animal Center of Tongji Hospital, Hubei Province, China. Cartilage from the knee joint was cut into pieces and digested with 0.25% trypsin-EDTA solution at 37°C for 30 min, centrifuged (1,000 rpm for 5 min), and washed with PBS. Then, the pieces were digested with 0.25% collagenase II in DMEM/F12 at 37°C for 8 h. SW1353 human chondrocytes were purchased from the Institute of Life Science Cell Culture Center (Shanghai, China). Chondrocytes from digested rat cartilage and SW1353 human chondrocytes were cultured in DMEM/F12 containing 10% fetal FBS (Gibco, NY, United States), 100 U/ml penicillin, and 100 U/ml streptomycin (Sigma-Aldrich, St. Louis, MO, United States) at 37°C with 5% CO_2_. Primary rat chondrocytes were used in this experiment.

### Cell Viability Assay

Cell viability was estimated with the CCK-8 assay. Briefly, SW1353 cells were seeded in 96-well plates at a density of approximately 2 × 10^3^ cells/well and treated with different concentrations of SA (0, 0.1,1,5,10, and 20 mM) for 24 h; the cells were subsequently exposed to 10 ng/ml IL-1β with or without SA (0.1, 1, 10 mM) for 24 h. Then, 10 μL CCK-8 solution was added to each well, and the wells were incubated for another 1 h in the dark at 37°C in 5% CO_2_. The absorption was detected at 450 nm with a microplate reader.

### Western Blotting

To obtain total protein, the treated cells were lysed with radioimmunoprecipitation assay (RIPA) lysis and extraction buffer (Boster, China) supplemented with 1% protease inhibitor cocktail and 1% phosphatase inhibitor cocktail. To prepare the nuclear and cytoplasmic fraction, the cells were lysed with a nuclear and cytoplasmic protein extraction kit (Beyotime, China) according to the manufacturer’s instructions. The lysates were centrifuged at 12,000 × g for 45 min at 4°C. The supernatant was harvested, and the protein concentration was measured according to the instructions on the BCA Protein Assay Kit. Samples with equal amounts of protein (25 μg) were separated by SDS-polyacrylamide gel electrophoresis and electrotransferred onto 0.3-μm polyvinylidene difluoride membranes (Millipore, Billerica, MA). After blocking with 5% bovine serum albumin (BSA) dissolved in Tris-buffered saline with Tween (TBST) for 1 h at room temperature, the membranes were incubated overnight at 4°C with specific antibodies. The antibodies were diluted in 5% BSA dissolved in TBST. After washing, the blots were incubated with horseradish peroxidase–conjugated goat anti-rabbit or anti-mouse IgG secondary antibodies for 1 h at room temperature. Finally, the blots were visualized with an enhanced chemiluminescence reagent (Boster, Wuhan, China), and the band intensities were quantified by a Bio-Rad ImageLab 5.0 system.

### mRFP-GFP-LC3 Adenovirus Transfection and Autophagic Flux Analysis

Cells were transfected with mRFP-GFP-LC3-carrying adenoviral vectors (HanBio Technology, Shanghai, China) according to the instructions. GFP-positive and mRFP-positive staining represented early autophagic organelles, and GFP-negative and mRFP-positive staining represented acidified autophagolysosomes. Autophagic flux was detected on a Nikon C2+ laser scanning confocal microscope (Nikon America Inc., Melville, NY).

### Cellular Safranin O Staining

Primary rat chondrocytes were seeded in 24-well plates and stimulated with IL-1β after pretreatment with or without SA for 48 h. Then, the cells were fixed with 4% paraformaldehyde (Boster, China) for 20 min at room temperature and washed with PBS three times to remove the medium. After another wash with PBS, the cells were stained with the safranin O working solution (Boster, China) for 1 h at 37°C. The remaining dye was removed, and the cells were washed three times with PBS. Finally, the 24-well culture plate was scanned with an inverted light microscope.

### Cellular Toluidine Blue Staining

Primary rat chondrocytes were seeded in a 24-well culture plate and stimulated with IL-1β after pretreatment with or without SA for 48 h. Then, the cells were washed with PBS three times, fixed with 4% paraformaldehyde for 20 min at room temperature, washed with PBS again, and then incubated with toluidine blue staining reagent (Boster, China). After a 4-h incubation at room temperature, the remaining toluidine blue stain was removed, and the cells were washed with PBS three times. The 24-well culture plate was scanned under an inverted light microscope to record the changes in each well.

### Rat Model of Osteoarthritis

Eighteen 6-week-old male Sprague Dawley rats were procured from the Laboratory Animal Center of Tongji Hospital. The study was approved by the Institutional Animal Care and Use Committee. A rat model of OA was established by anterior cruciate ligament transection (ACLT) surgery on the right knees. The control group underwent a sham operation. One week after the surgery, the OA model rats were treated with intra-articular injection of SA (20 mM in 100 µL) or vehicle twice a week for one month. One month post-SA treatment, the rats were sacrificed, and the knee joints from the rats were fixed with 4% paraformaldehyde for 48 h.

### Histological Evaluation

The rat joint tissues were routinely decalcified with 10% EDTA for 6–8 weeks, embedded in paraffin, and sectioned at a thickness of 5 µm. Then, the sections were stained with toluidine blue and safranin O/fast green. The semiquantitative histological grading criteria of Kraus’ modified Mankin’s score and the Osteoarthritis Research Society International (OARSI) scoring system were used to evaluate the histopathological changes, and the scores were consistent with the progression of OA (Mankin and Lippiello, 1970; Gerwin et al., 2010).

### Statistical Analysis

The results are shown as the mean ± standard deviation (SD). All the experiments were repeated at least three times. Data from three or more groups were compared by one-way analysis of variance (ANOVA) followed by Tukey’s post hoc test; unpaired Student’s t test was used to compare two groups; and nonparametric data (OARSI scores) were analyzed by the Kruskal–Wallis H test. *p*-Values< 0.05 were considered to indicate significant differences.

## Results

### Shikimic Acid Has No Impaired Effect on Cellular Viability and 10 mM Concentration Was Utilized in Subsequent Experiments

**FIGURE 1:**
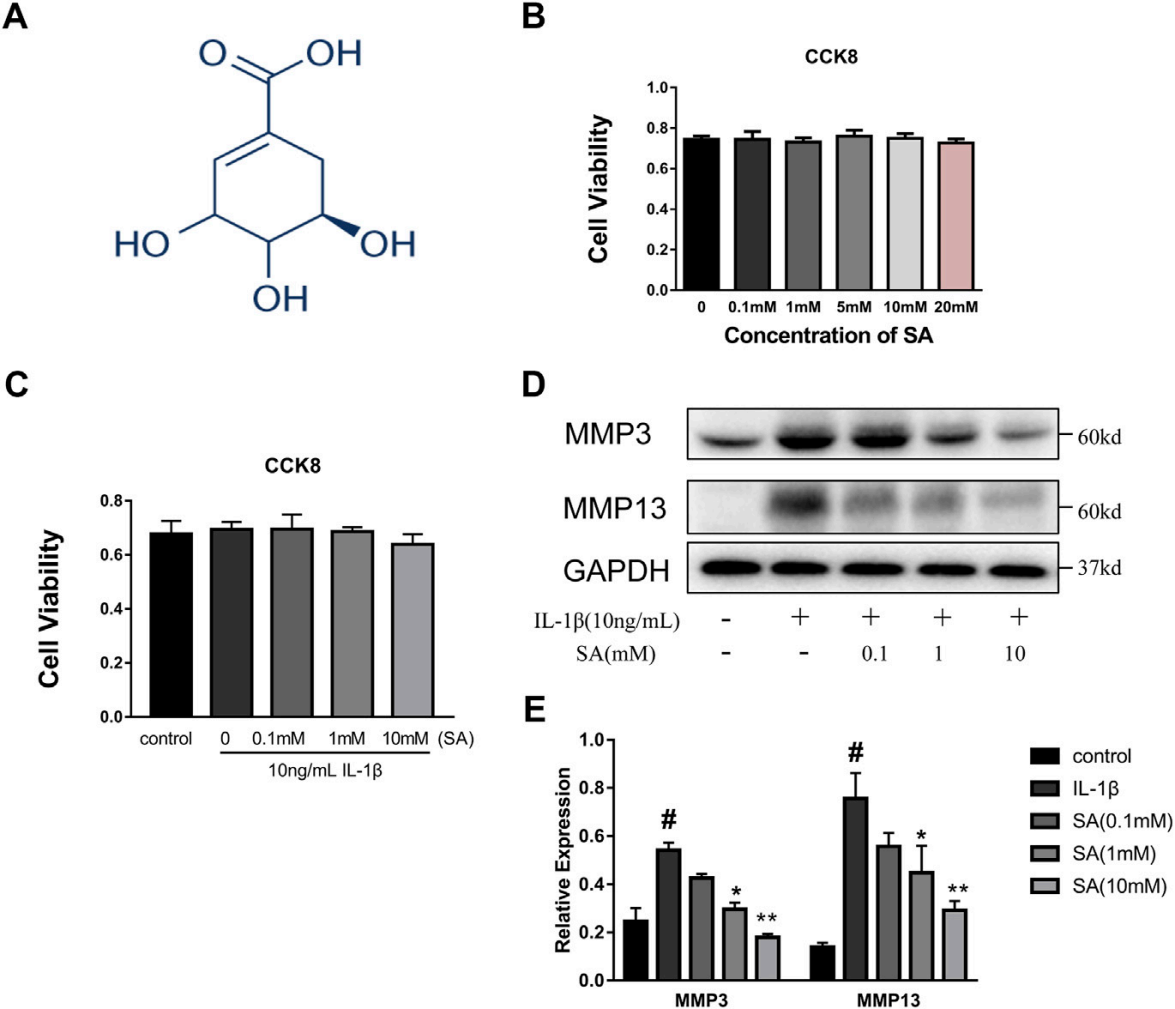
SA has no impaired effect on cellular viability and 10 mM concentration is utilized in subsequent experiments. **(A)** Chemical structure of SA; **(B)** SW1353 cells were exposed to increasing concentrations of SA (0, 0.1, 1, 5, 10, and 20 mM), and cell viability was determined by CCK-8 assay. The data are shown as the mean ± SD (*n* = 5); **(C)** SW1353 cells were exposed to 10 ng/ml with or without SA, and cell viability was determined by the CCK-8 assay. The data are shown as the mean ± SD (*n* = 5). **(D, E)** Representative western blots and quantification data showing the MMP3 and MMP13 expression in the SW1353 cells of each group. The data are shown as the mean ± SD (*n* = 3). Significant differences between groups are indicated as follows: ^#^ *p* < 0.05 vs. the control group; *p < 0.05 and **p < 0.01 vs. the IL-1β group.

A CCK8 assay was used to assess the cytotoxic effects of SA on SW1353 cells. The cells were cultured with increasing concentrations of SA (0, 0.1, 1, 5, 10, and 20 mM). As shown in Figure 1B, incubation with SA for 24 h resulted in no significant difference in cell viability compared with that of the control group (*p* > 0.05). Cells were also exposed to 10 ng/ml IL-1β with or without SA, and the CCK-8 results showed that 10 ng/ml IL-1β with or without SA (0.1, 1, and 10 mM) had no effect on cell viability (Figure 1C). Increasing concentrations of SA (0.1, 1, and 10 mM) were used for the next experiment. SW1353 cells pretreated with or without SA for 1 h were stimulated with IL-1β (10 ng/ml) for 24 h and then harvested for sample extraction and evaluation of the effects of SA on the IL-1β-induced increasing of MMP3 and MMP13. As shown in Figures 1D,E, SA at a concentration of 10 mM notably alleviated the IL-1β-induced expression of MMP3 and MMP13; 10 mM of SA was thus utilized in the following experiments.

### Shikimic Acid Ameliorates the IL-1β-Induced Expression of iNOS and COX2 in SW1353 Cells

**FIGURE 2:**
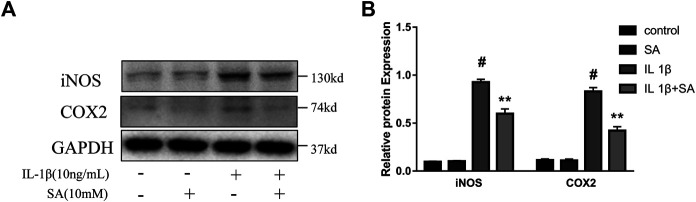
SA ameliorates IL-1β-induced expression of iNOS and COX2 in SW1353 cells. Representative western blots and quantification data showing iNOS and COX2 expression in cells treated as described above. The data are shown as the mean ± SD (*n* = 3). ^#^ *p* < 0.05 vs. the control group; **p < 0.01 vs. the IL-1β group.

To evaluate whether SA could inhibit the increase of iNOS and COX2 induced by IL-1β, SW1353 cells were exposed to IL-1β after pretreatment with or without SA for 24 h. And the western blot was used to evaluate the effects of SA on iNOS and COX2 expression. The results showed that SA inhibited the IL-1β-stimulated increases in iNOS and COX2 protein expression (Figure 2).

### Shikimic Acid Attenuates IL-1β-Stimulated Anabolic and Catabolic Dysregulation in SW1353 Cells

**FIGURE 3:**
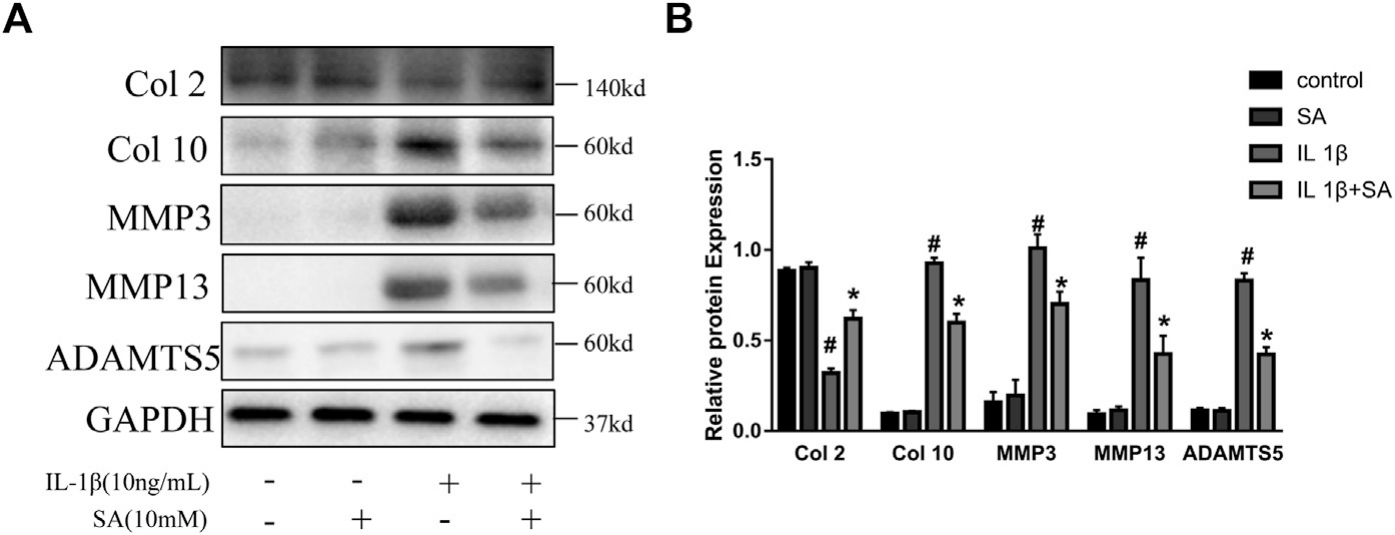
SA attenuates IL-1β-stimulated anabolic and catabolic dysregulation in SW1353 cells. Representative western blots and quantification data showing the Col 2, Col 10, MMP3, MMP13, and ADAMTS5 expression in cells treated as described above. The data are shown as the mean ± SD (*n* = 3). ^#^ *p* < 0.05 vs. the control group; *p < 0.05 vs. the IL-1β group.

During the progression of OA, MMPs (MMP3 and MMP13) and ADAMTS5 are the major enzymes involved in catabolic dysregulation, while Col 2 is involved in anabolism (Cui et al., 2017). The expression of Col 10 is one of the major characteristics of chondrocyte hypertrophy, which is followed by cartilage matrix degradation and vascular invasion (Moskowitz, 2000). In this study, cells were stimulated with IL-1β after pretreatment with or without SA for 24 h. As shown in Figure 3, IL-1β could significantly increase the expression of MMP3, MMP13, ADAMTS5, and Col 10 but reduced the expression of Col 2, and SA reversed the changes.

### Shikimic Acid Ameliorates IL-1β-Induced Impairment of Autophagy in SW1353 Cells

**FIGURE 4:**
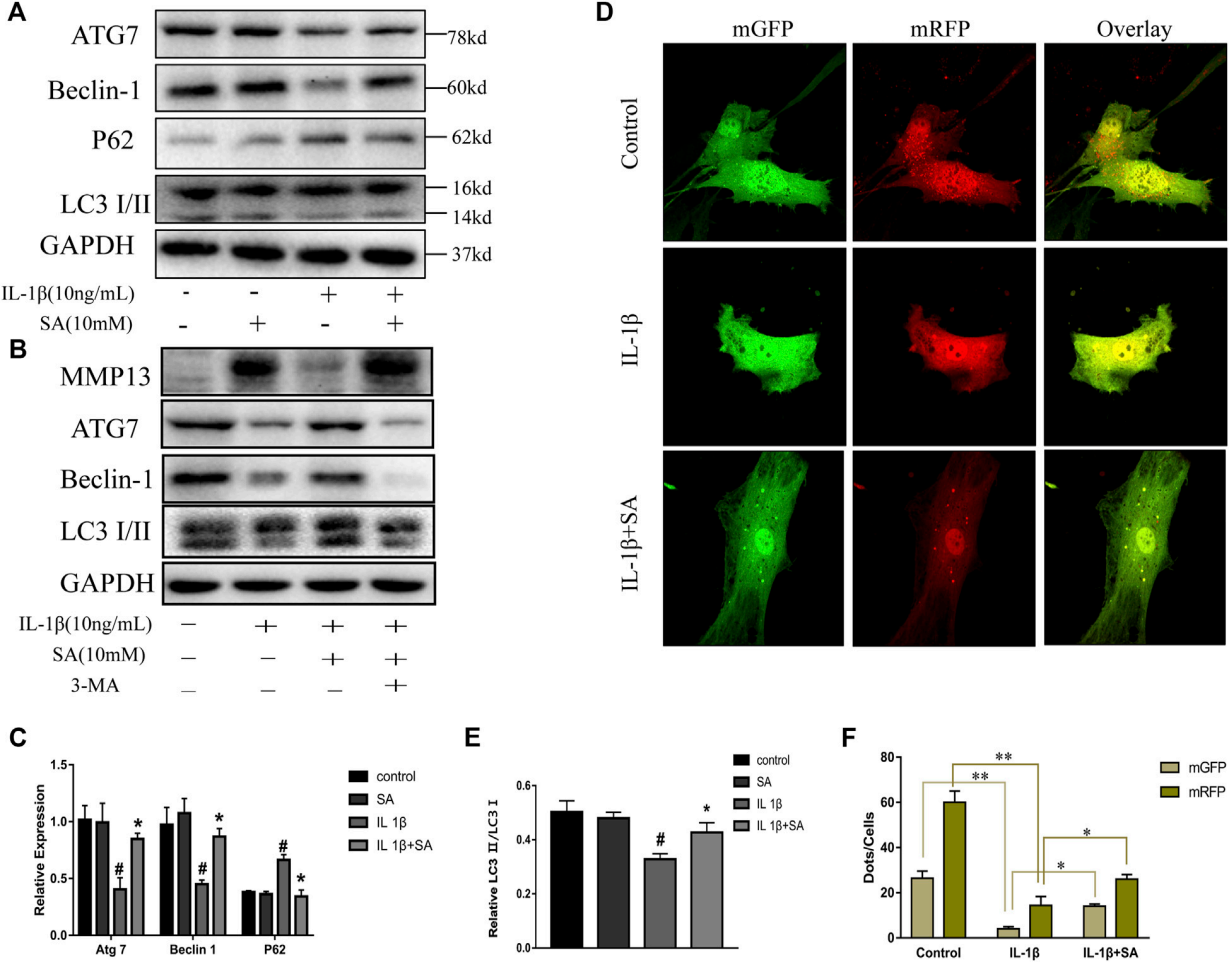
SA ameliorates IL-1β-induced impairment of autophagy in SW1353 cells. **(A,C,E)** Representative western blots and quantification data showing ATG7, Beclin-1, P62 and LC3 expression in cells treated as described above. The data are shown as the mean ± SD (*n* = 3). ^#^ *p* < 0.05 vs. the control group; **p < 0.01 vs. the IL-1β group. **(B)** Western blot showing the MMP13, ATG7, Beclin-1, and LC3 expression in cells treated as described above. **(D,F)** Fluorescence microscopy analysis of chondrocytes transfected in each group with mRFP-GFP-LC3-carrying adenovirus. The data are shown as the mean ± SD (*n* = 3). Significant differences between groups are indicated as **p < 0.01, *p < 0.05.

Previous studies have indicated that autophagy is involved in the progression of OA and plays an important role in preventing the degeneration of cartilage (Luo et al., 2019). Different levels of autophagic activity have been observed in different sections of OA cartilage, and low levels of autophagy have been found in OA patients; notably, autophagy stimulation and autophagic flux restoration protect against the development of OA (Jeon and Im, 2017; Tang et al., 2017). Here, to determine whether SA affects IL-1β-induced autophagy impairment, cells were exposed to IL-1β after pretreatment with or without SA for 24 h and autophagy-related molecules were detected. As indicated in Figure 4A, IL-1β treatment reduced ATG7 and Beclin-1, reduced the ratio of LC3-II to LC3-I, and induced P62 accumulation, suggesting that it impaired autophagy in chondrocytes. However, SA alleviated this impairment. To confirm the effect of SA on the autophagic flux of IL-1β-stimulated cells, a tandem GFP-RFP-LC3-carrying adenovirus was used to measure the autophagic flux (Kaizuka et al., 2016). A decrease in GFP indicates the fusion of lysosome and autophagosomes to form autophagosomes, and red spots indicate autophagy lysosomes. The level of autophagic flux can be clearly determined via quantification of different colored spots. As shown in Figure 4D, SA treatment restored autophagic flux in the IL-1β-stimulated cells. To investigate the involvement of SA in IL-1β-induced autophagy impairment and cartilage degradation, 3-methyladenine (3-MA), a specific autophagy inhibitor, was used before SA treatment. Cells pretreated with 3-MA (at 5 mM, Selleck Chemicals, Houston, TX, United States) for 2 h were stimulated with IL-1β with or without pretreatment of SA for 24 h. As shown in Figure 4B, compared with the IL-1β-stimulated group without SA pretreatment, SA treatment decreased the expression of MMP13, increased the expression of Beclin-1, and restored the conversion from LC3-I to LC3-II. However, 3-MA reversed the effect of SA.

### Shikimic Acid Restraints IL-1β-Induced Activation of the MAPK Signaling Pathway

**FIGURE 5:**
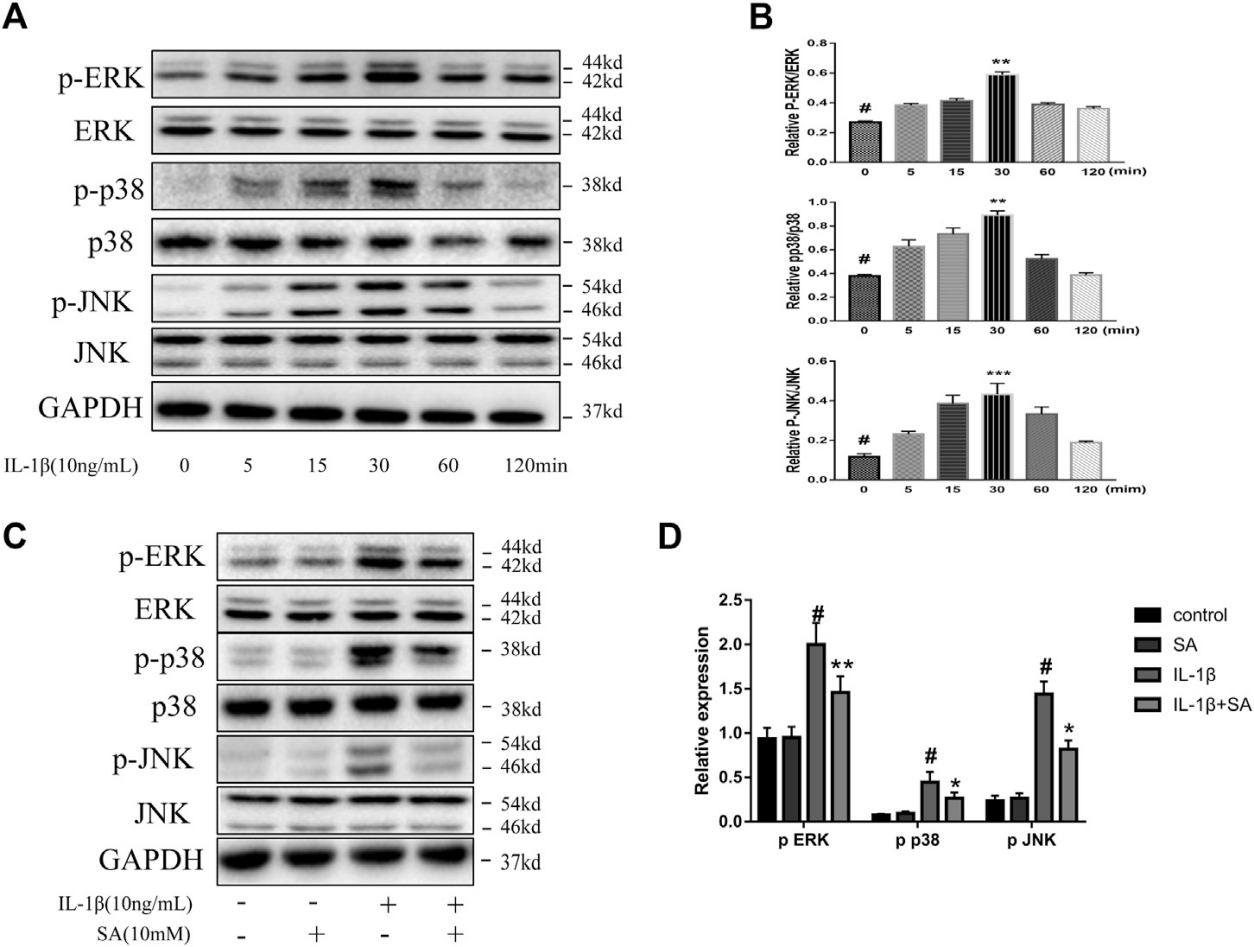
SA restrain IL-1β-induced activation of the MAPK signaling pathway. **(A,B)** Representative western blots and quantification data showing p-ERK, ERK, p-p38, p38, *p*-JNK and JNK expression in cells treated with IL-1β for different time points. The data are shown as the mean ± SD. ***p < 0.001, **p < 0.01 vs **#** the control group (*n* = 3). **(C,D)** Representative western blots and quantification data showing *p*-ERK, ERK, p-p38, p38, *p*-JNK, and JNK expression in cells after 30 min of IL-1β exposure. The data are shown as the mean ± SD (*n* = 3). ^#^ *p* < 0.05 vs. the control group; **p < 0.01, *p < 0.05vs. the IL-1β group.

The MAPK signaling pathway is significantly related to the progression of OA, and MAPK is a mediator which regulates the downstream expression of proinflammatory cytokines and MMPs (Xie et al., 2019; Chow and Chin, 2020). A previous study showed that the MAPK signaling pathway is activated within a short time after IL-1β exposure (Liacini et al., 2002). Accordingly, we investigated the effect of SA on the MAPK signaling pathway during IL-1β exposure. SW1353 cells were stimulated with IL-1β and harvested at 0, 5, 15, 30, 60, and 120 min for analysis. We found that the levels of phosphorylated JNK, ERK, and P38 rapidly peaked within 30 min after IL-1β exposure (Figures 5A,B). Cells pretreated with or without SA were stimulated with IL-1β for 30 min. As shown in Figures 5C,D, SA effectively suppressed the IL-1β-induced increases in p-ERK, p-p38, and p-JNK levels, suggesting that inhibition of IL-1β-induced MAPK activation might be involved in the protective effect of SA against cartilage degradation.

### Shikimic Acid Mitigates IL-1β-Induced Activation of the NF-κB Signaling Pathway

**FIGURE 6:**
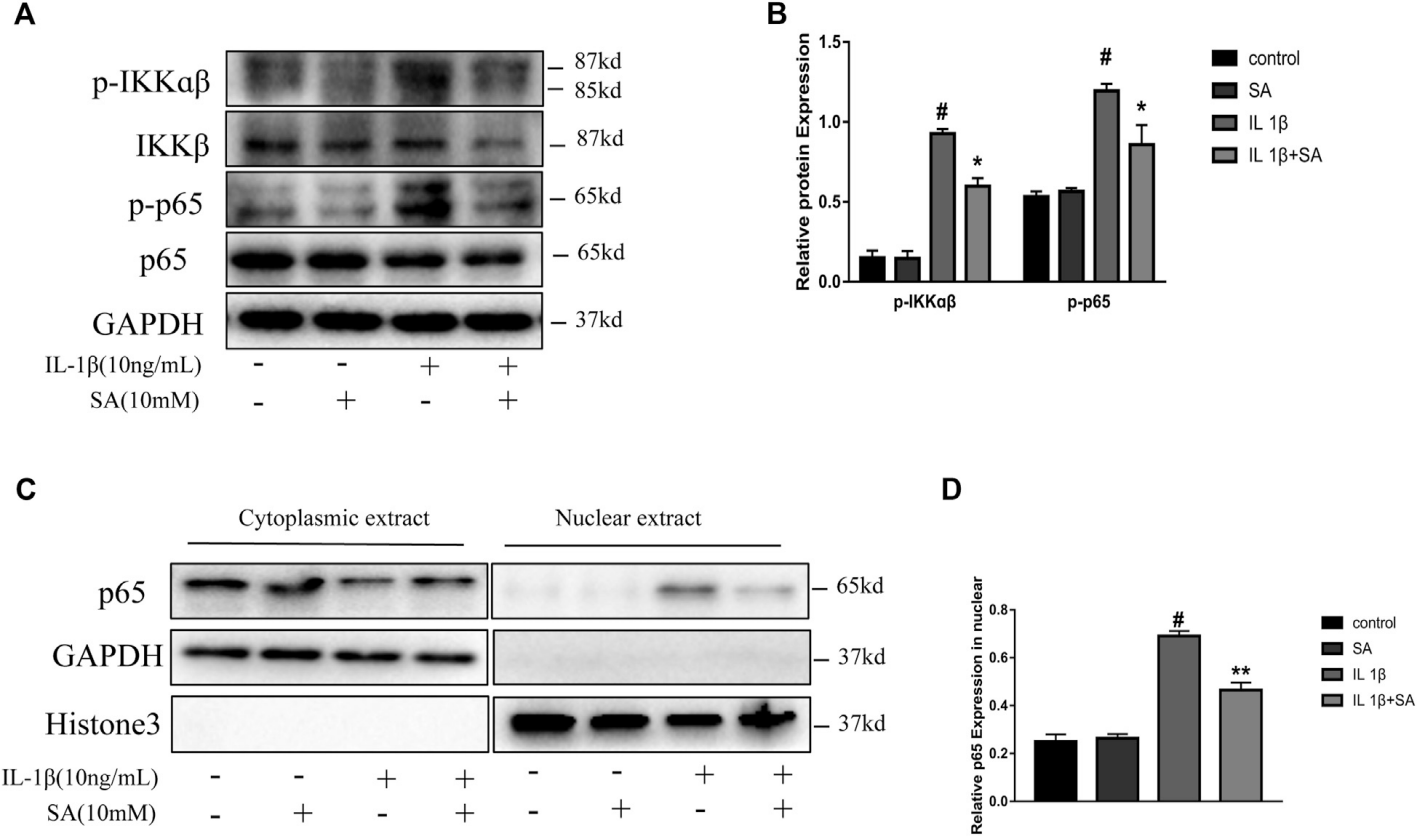
SA mitigates IL-1β-induced activation of the NF-κB signaling pathway. **(A,B)** Representative western blots and quantification data showing *p*-IKKα/β, IKKα/β, p-p65, and p65 expression in cells treated as described above. **(C,D)** Representative western blots and quantification data showing cytoplasmic P65 and nuclear P65 levels in cells treated as described above. The data are shown as the mean ± SD (*n* = 3). ^#^ *p* < 0.05 vs. the control group; **p < 0.01, *p < 0.05vs. the IL-1β group.

The NF-κB signaling pathway plays a vital role in the inflammation response and cartilage degeneration in OA (Choi et al., 2019). NF-κB signaling pathway activation induces catabolic gene expression through NF-κB response elements located in the promoters of the MMP and ADAMTS5 genes and promotes the expression of major pro-inflammatory and destructive mediators of OA, including COX2 and iNOS (Bond et al., 1998; Lianxu et al., 2006; Ulivi et al., 2008; Kobayashi et al., 2013). In general, the NF-κB dimers are retained in the cytoplasm through their interaction with inhibitory IκB proteins. After stimulation, IκB proteins are phosphorylated by IκB kinases (IKKs) and degraded, allowing NF-κB complexes to translocate to the nucleus and transactivate the expression of proinflammatory cytokines (Hayden and Ghosh, 2008). In this study, the IL-1β-induced phosphorylation of IKKα/β and p65 were effectively suppressed by pretreatment with SA (Figures 6A,B). Moreover, SA inhibited the IL-1β-induced translocation of the p65 subunits into the nucleus in SW1353 chondrocytes (Figure 6C). The results above indicate that suppression of IL-1β-stimulated NF-κB activation might be involved in the protective effect of SA against cartilage degeneration.

### Shikimic Acid Mitigates Cartilage Degradation in Rat Chondrocytes *In Vitro* and a Rat Osteoarthritis Model *In Vivo*

**FIGURE 7:**
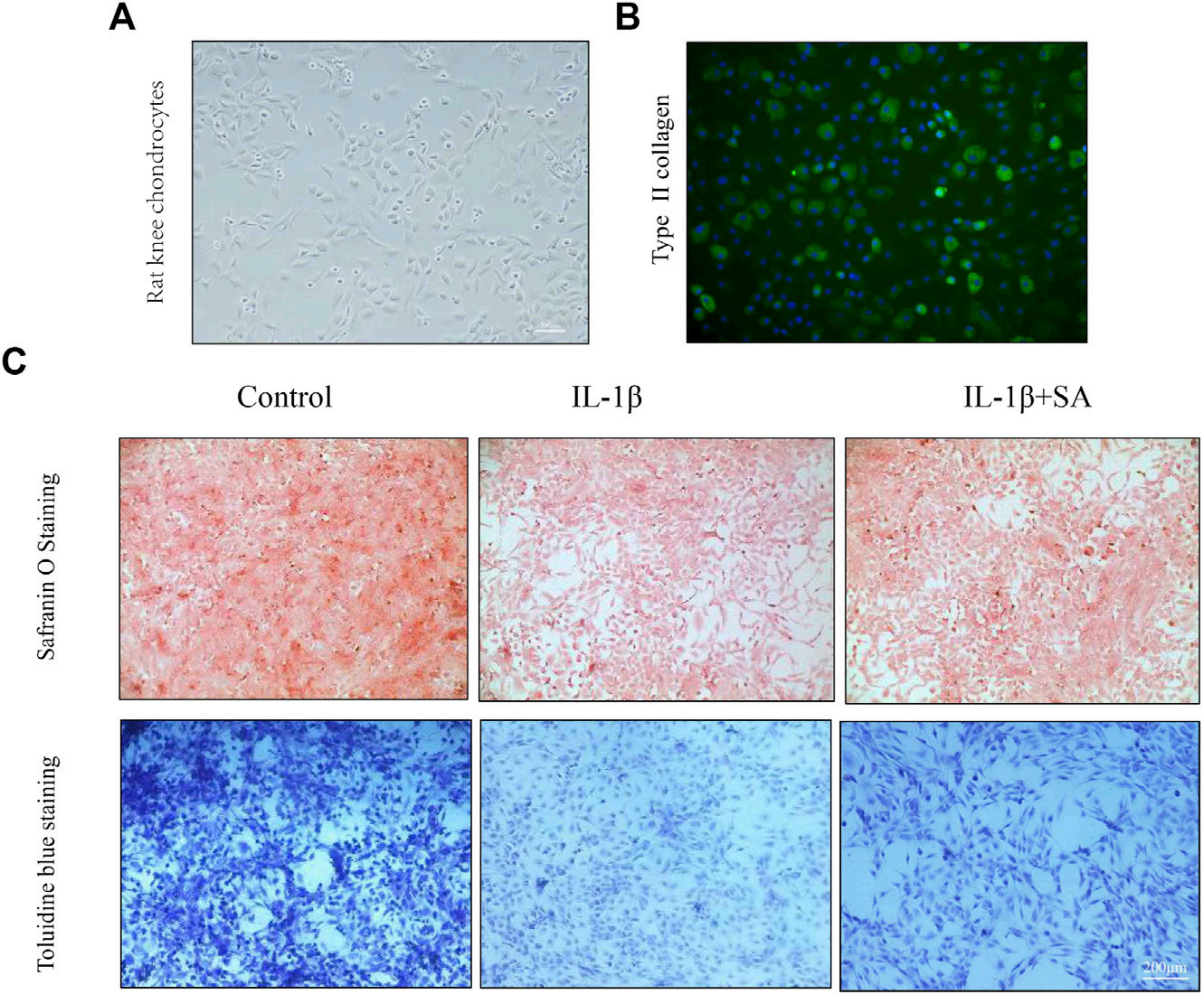
SA mitigates cartilage degradation of ECM of rat knee chondrocytes *in vitro*. **(A)** Phase-contrast micrographs of rat knee chondrocytes plated on culture dishes. **(B)** Col 2 immunofluorescence staining of rat knee chondrocytes. **(C)** Safranin O and toluidine blue staining for proteoglycans deposition in each group.

**FIGURE 8:**
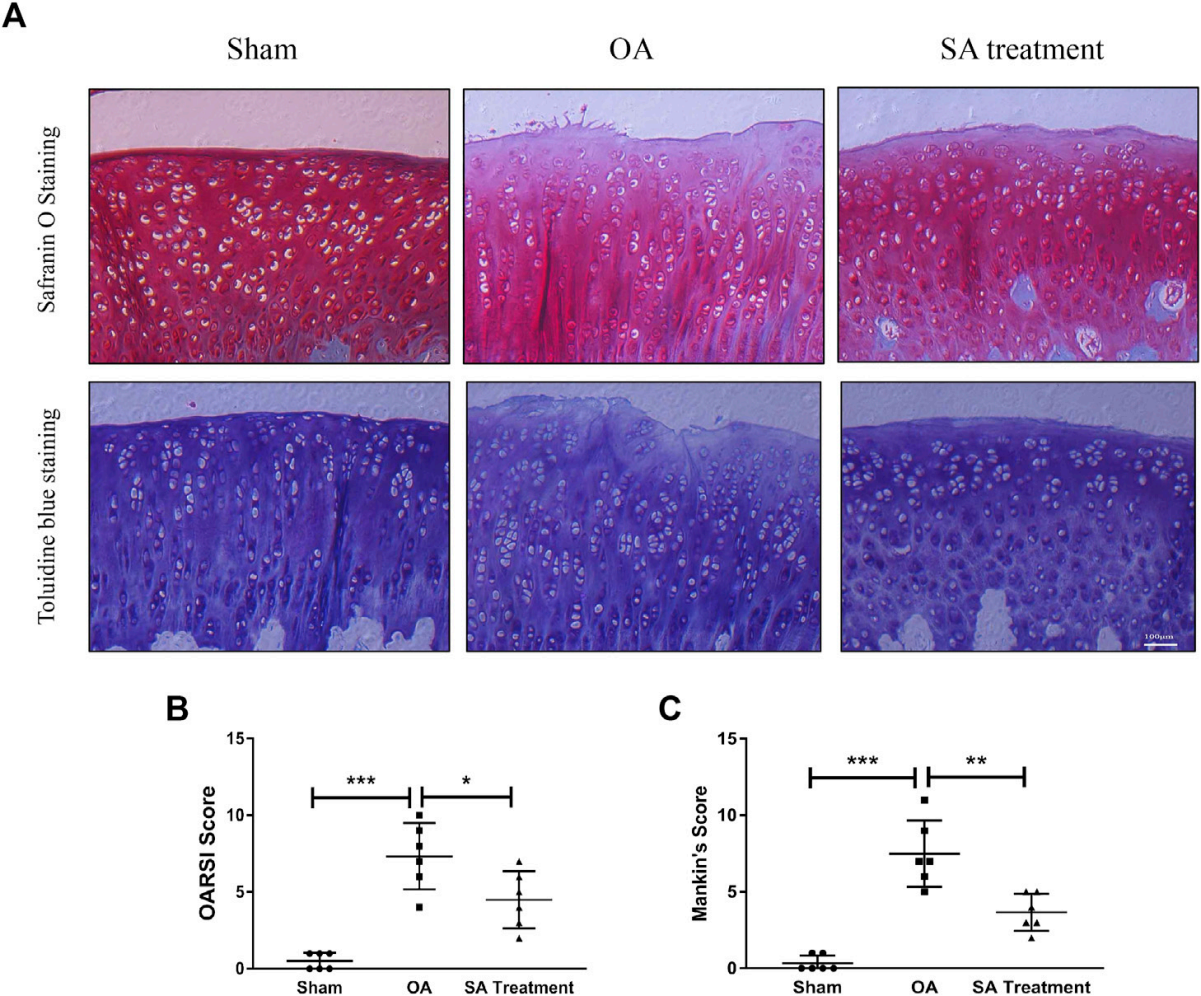
SA mitigates cartilage degradation in a rat OA model *in vivo*. **(A)** Microscopic photos of safranin-O/fast green- and toluidine blue-stained sections of rat knee joints from the three groups. **(B)** OARSI scores of each group. The data are shown as the mean ± SD (*n* = 6). Significant differences between groups are indicated as ***p < 0.001; ∗*p* < 0.05.

Safranin O staining and toluidine blue staining are commonly used to confirm the degeneration of cartilage. Safranin O interacts with the cartilage matrix and the synthetic catabolic activity of cartilage can be analyzed based on the different intensities of red staining (Kahveci et al., 2000). Safranin O without fast green is considered to be the best staining agent for evaluating the degradation of macromolecules in the cartilage ECM (Alibegović et al., 2020). Toluidine blue is a classical staining method applied to cartilage, and it is generally accepted that the degree of positive staining corresponds to the amount of proteoglycans (Bergholt et al., 2019). To assess the therapeutic effects of SA on IL-1β-induced cartilage degradation in the early stage *in vivo*, rat knee primary chondrocytes and knee cartilage of OA model rats were stained with safranin O and toluidine blue. In our study, severe cell shrinkage and ECM degradation were observed in rat primary chondrocytes stimulated with IL-1β for 48 h, but SA significantly attenuated IL-1β-associated degeneration and matrix disruption (Figure 7C). In addition, to evaluate the effects of SA on rats with OA *in vivo*, we established a rat model of OA *via* ACLT surgery. After a month of intra-articular injection of SA, knee joint tissues were collected from the three groups (the sham, OA model, and SA treatment groups). Complications or infections were not observed in any of the animals during the experiment. The tissues were stained with toluidine blue and safranin O/fast green for histological evaluation. As shown in Figure 8A, we observed clear articular cartilage erosion and loss of safranin O staining and toluidine blue staining in the OA model group compared with the sham group. Moreover, the OARSI scores and Mankin’s scores showed that the SA treatment alleviated the progression of OA (Figures 8B,C).

## Discussion

**FIGURE 9:**
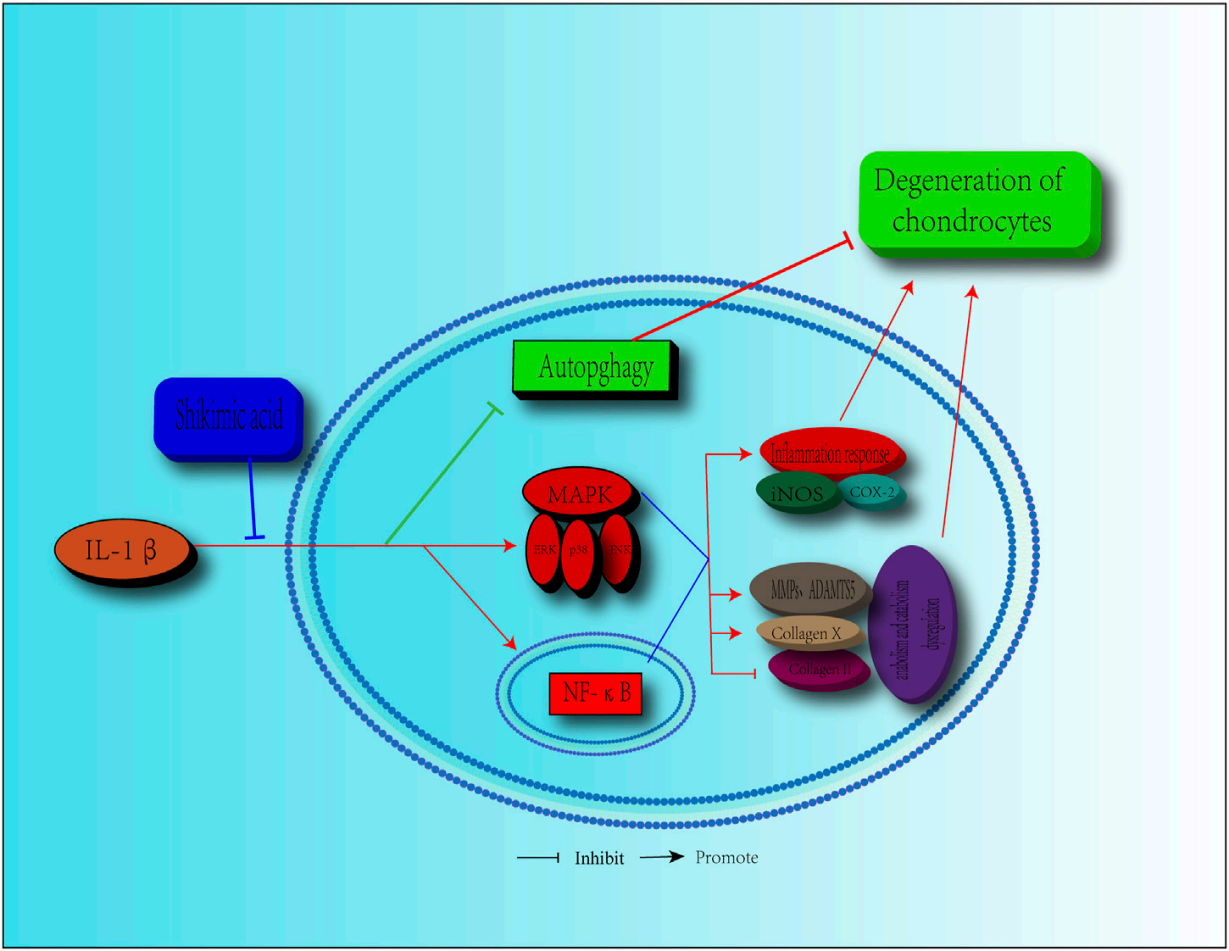
Schematic diagram of the effect of SA on cartilage degeneration. IL-1β induces the expression pro-inflammatory factors, including iNOS, COX2, MMPs, and ADAMTS5. IL-1β stimulates the increasing of collagen X, degradation of collagen II and impairment of autophagy. Furthermore, IL-1β functions by activating the MAPK and NF-κB signaling pathways, as shown by, the increase in the level of *p*-ERK, p-p38, *p*-JNK, *p*-IKKα/β, p-p65, and the translocation of p65. However, SA can reverse these effects.

OA, a degenerative disease that is common in the elderly population, is a major cause of cartilage pain and limited mobility (Martel-Pelletier et al., 2016; GBD 2016 DALYs and HALE Collaborators, 2017). As the population ages, more people suffer from OA (McAlindon et al., 2014). However, the current treatments are limited, focusing mainly on symptom relief via control of the inflammatory response and pain, as the pathological progression of OA is not well understood (Martel-Pelletier et al., 2016). Artificial joint replacement surgery is the only choice for most patients with OA. Therefore, alternative and effective agents for OA treatment are urgently needed. SA, which is extracted from *Illicium verum*, is a natural ingredient that shows a potential value for disease treatment due to its anti-inflammatory effects (Wang et al., 2011; Ghosh et al., 2012). In this study, we revealed that SA exerted protective effects against IL-1β-induced inflammation and cartilage degeneration via activation of the MAPK/NF-κB signaling pathway and restoration of impaired autophagy in chondrocytes (Figure 9).

Degradation of the ECM of articular chondrocytes is the most important process in the development of OA. Under normal conditions, the regulation of anabolism and catabolism plays a key role in maintaining the balance of the ECM (Hunter, 2014). Inflammatory factors, especially IL-1β, are widely considered to be a major cause of ECM metabolic disorders, and IL-1β is commonly used to establish *in vitro* models of OA (Fukui et al., 2003; Caramés et al., 2008). The human chondrosarcoma cell line SW1353, initiated in the 1970s, has similarities respect to catabolic effects after IL-1β exposure compare with articular chondrocytes, and SW1353 is considered to be a valuable *in vitro* system for investigating the regulation of catabolic genes by IL-1β (Liacini et al., 2003; Schaefer et al., 2003; Gebauer et al., 2005). Here, we chose 10 ng/ml IL-1β for the experiments conducted in SW1353 cells and rat knee chondrocytes according to previous research (Mao et al., 2018; Li et al., 2020). Our results indicated that IL-1β significantly increased the expression of inflammatory factors, including iNOS and COX2. This increase was accompanied by the increasing of MMP3, MMP13, and ADAMTS5 and the reduction of Col 2, which contributed to the imbalance of anabolism and catabolism in the chondrocytes. In addition, the expression Col 10, one of the major factors involved in chondrocyte hypertrophy (which is followed by cartilage matrix degradation), was increased by IL-1β. The safranin O staining and toluidine blue staining results indicated that severe cell shrinkage and ECM degradation occurred in rat primary chondrocytes stimulated with IL-1β. Furthermore, we found that IL-1β impaired autophagy, leading to anabolic and catabolic dysregulation via the reduction of ATG7 and Beclin-1 expression, reduction of the LC3-II to LC3-I ratio, accumulation of P62 and inhibition of the autophagic flux. All these changes were alleviated by SA. Taken together, these results show that SA relieves cartilage degeneration *in vitro*. *In vivo*, we established rat models of OA to further determine the protective effects of SA against cartilage degradation, and histological evaluation confirmed the potential therapeutic effects of SA mediated by the amelioration of cartilage degradation and destruction.

The MAPK and NF-κB signaling pathways are critical during the occurrence and development of OA (Saklatvala, 2007; Wong et al., 2020). Activation of the MAPK pathway contributes to cartilage degradation induced by anabolic and catabolic dysregulation in the cartilage ECM (Malemud, 2017). The transcription factor NF-κB is associated with various inflammatory responses that lead to OA, and NF-κB has been considered a promising therapeutic target in OA (Zhou et al., 2019). Activation of the MAPK and NF-κB signaling pathways leads to MMPs and ADAMTS 5 overexpression and inflammatory responses, which further cause degradation of the cartilage matrix; in addition, the inhibition of the MAPK or NF-κB pathways can directly or indirectly alleviate the progression of OA by inhibiting the expression or activity of OA-associated factors (Malemud et al., 2003; Roman-Blas and Jimenez, 2006; Sondergaard et al., 2010). Moreover, suppression of NF-κB via inhibitors and knockdown can relieve the impairment of autophagy (Chen et al., 2020). The MAPK signaling pathway can suppress autophagy (Chen and Klionsky, 2011), but it may also regulate in the progression of OA in an autophagy-independent manner (Zhou et al., 2015). Further research is required to test this hypothesis. In this study, IL-1β activated the MAPK and NF-κB pathways by clearly promoting the phosphorylation of ERK, p38, JNK, p65, and IKKα/β as well as the nuclear translocation of p65. Nevertheless, these changes could be reversed by SA.

The ACLT rat model is a versatile and widely used to analyze the progress of OA (Lorenz and Grässel, 2014). In this study, the ACLT group showed apparent cartilage erosion and proteoglycan loss, while the SA treatment group exhibited partially alleviation of these changes, indicating that SA treatment might delay the progression of OA *in vivo*. However, we performed intra-articular injections of SA at one week after ACLT surgery and harvested joint samples immediately after four weeks of injections; thus, only a relatively short-term efficacy during the early phase of posttraumatic OA could be observed. The long-term effects of SA need further investigation.

In conclusion, our results reveal that SA can attenuate the IL-1β-induced inflammatory response, anabolic and catabolic dysregulation in cartilage, and impairment of autophagy in chondrocytes and that the MAPK and NF-κB signaling pathways might be involved in this protective effects. These findings suggest that SA may be a new potential strategy for the treatment of OA.

## Data Availability

The raw data supporting the conclusion of this article will be made available by the authors, without undue reservation.
